# A CFD-based investigation of hydraulic regimes in overflow weir fishways

**DOI:** 10.1371/journal.pone.0345694

**Published:** 2026-09-25

**Authors:** Xu Wang, Xiangkun Yang, Fan Wang, Na Xu, Yicai Teng, Zheng Qin, Ping Yu

**Affiliations:** 1 College of Water Conservancy Engineering, Tianjin Agricultural University, Tianjin, China; 2 Beifang Investigation, Design and Research Co., Ltd., Tianjin, China; UTRGV: The University of Texas Rio Grande Valley, UNITED STATES OF AMERICA

## Abstract

Overflow weir fishways represent a basic type of pool-and-weir fishway structure. To investigate its hydraulic characteristics, this study conducts numerical simulations to obtain the flow field in a typical pool chamber. A total of nine cases are designed by varying the inlet discharge and pool depth, and the flow patterns, velocity distribution, turbulent kinetic energy, and dissipation rate are systematically analyzed. Three flow regimes are identified: plunging flow, streaming flow, and transition flow. The variation laws of these regimes with inlet discharge and pool depth are obtained. The distributions and evolution characteristics of velocity, turbulent kinetic energy, and dissipation rate are also summarized. The results show that the flow regime is jointly controlled by inflow discharge and vertical drop between adjacent pools. With the increase of inflow discharge, the flow regime shifts from plunging flow to transition flow and then to streaming flow. Under the same inflow discharge, a larger vertical drop tends to form plunging flow with lower turbulence intensity. This study reveals the fundamental hydraulic characteristics of overflow weir fishways and provides a reference for the structural design and hydraulic optimization of such fishways.

## 1. Introduction

Fishway is a fish-passing structure constructed at barriers such as dams to restore fish migration routes. It serves as a biological corridor, facilitating the exchange of matter and energy between upstream and downstream sections [1]. Upon entering the pool, water in the fishway dissipates energy through diffusion and reentry processes, generating flow patterns, turbulence levels, and vorticity conducive to fish migration [2]. The velocity and depth of flow in a fishway are influenced by the design of the fishway, the slope of the channel, and the discharge rate [3]. The fishway is a crucial engineering infrastructure that uses a unique structure to disperse energy and create favorable water flow conditions in order to conserve fish resources [4].

The overflow weir fishway is one of the most basic forms of baffle fishways. Fish leap over the weir crest from the upstream side, with baffles arranged in a stepped configuration along the bottom slope. In order for the fishways located upstream to efficiently and successfully aid the movement of fish from one point to another, a multitude of specific requirements and conditions must be met and fulfilled in a meticulous and precise manner [5]. Water flows over the weir and dissipates energy through the downstream plunge pool. Although this type of fishway offers effective energy dissipation, its overflow capacity is relatively limited, and its adaptability to fluctuating upstream and downstream water levels is somewhat limited. The turbulence level in the flow field increases with discharge. Owing to its sensitivity to small changes in inflow discharge and tailwater elevation, the overflow weir fishway has clear limitations in application scenarios. This sensitivity is a key disadvantage in waters with frequent water level fluctuations, as it will break the stable flow structure required for fish passage; however, it also confirms that the overflow weir fishway is most suitable for projects with stable tailwater conditions, which is a non-negligible design consideration for this type of fishway.

With the rapid advancement of science and technology, computational capabilities have significantly improved. Numerical simulation techniques have long been employed in hydraulic engineering, and computational fluid dynamics has been extensively studied across disciplines. Numerical simulation offers advantages such as low experimental cost, high computational efficiency, and reliable results. It effectively complements physical model experiments and supports practical engineering solutions. This reinforces the feasibility of using simulation to analyze flow fields under various operating conditions and to optimize fishway design based on fish migration behavior. In recent years, numerous researchers worldwide have applied numerical methods to study fishway hydraulics, aiming to enhance fishway efficiency and provide optimization criteria and specific recommendations for structural design. Leschziner and Rodi [6] achieved satisfactory results by simulating open-channel flow using the k-ε model. Hirt and Nichols [7] developed the Volume of Fluid (VOF) method based on a marker grid approach, enabling the simulation of free-surface flows [8]. numerically simulated the flow field over a spillway using the VOF method and the standard k-ε model, validating results with physical model tests. Hong Fangwen [9] applied the VOF method to simulate flow around moving bodies near a free surface, proposing an improved pressure interpolation scheme and demonstrating the method’s potential. Cui Zhanfeng [10] employed the RNG k-ε turbulence model coupled with a flow and bedload transport coupled model to calculate the scouring patterns of spur-dikes. The results were compared with field measurement data, demonstrating that this model can be used to simulate scouring and siltation changes in terrain near spur-dikes. Huang Haiyan [11] established a model of a sluice gate with a small opening, simulated the flow in the gate slot using VOF and RNG k-ε models, and verified the model’s reliability. Liu Hu et al. [12] employed the RNG k-ε model and VOF method to analyze the hydraulics of a combined vertical-slot and orifice fishway, providing references for design optimization. Li Lie et al. [13] validated a fishway model design by simulating flow patterns and turbulent kinetic energy using the RNG k-ε model, TruVOF method, and FAVOR technique, offering technical support for practical construction. Jiang Yongqiang [14] computed the flow field in a vertical-slot fishway using the RNG k-ε model, with results showing a mean error of about 10%, confirming the model’s practical utility. Marco Tiberga et al. [15] proposed a Discontinuous Galerkin (DG) algorithm to solve RANS equations with the k-ε model, improving the accuracy of Reynolds-averaged Navier–Stokes solutions. Shi Xunlei et al. [16] used Flow-3D software to simulate flow characteristics at different fishway inlet positions and orientations, optimizing inlet layout based on velocity and turbulent energy analysis to enhance fish passage.

In this study, numerical simulation was conducted to investigate the hydraulic characteristics of an overflow weir fishway by varying pool water depth and inlet discharge. Flow patterns, velocity, turbulent kinetic energy, and turbulent dissipation rate were systematically analyzed. The results provide a reference for selecting basic fishway types and designing new fishways.

## 2. Mathematical model and verification

### 2.1. Governing equations of the turbulence model

Fluid motion follows the conservation laws of mass, momentum, and energy. Thus, turbulent flow can be described by the continuity equation, Navier–Stokes equations, and energy equation. To close the governing equations, turbulence models approximate higher-order unknown correlation terms with lower-order correlations or time-averaged variables. The RNG k-ε model is derived from the instantaneous Navier–Stokes equations using renormalization group theory.

The continuity equation is as follows:


$$\begin{array}{c} \frac{\partial u_{i}}{\partial x_{i}}=0 \end{array}$$ (1)

The Navier-Stokes equation is as follows:


$$\begin{array}{c} \frac{\partial u_{i}}{\partial t}+u_{j}\frac{\partial u_{i}}{\partial x_{j}}=\;-\text{\textbackslash{}hspace\{0.17em}\}\frac{\text{1}}{\rho }\frac{\partial p}{\partial x_{i}}+\nu \frac{\partial^{\text{2}}u_{i}}{\partial x_{i}\partial x_{j}}+g_{i} \end{array}$$ (2)

The energy equation is as follows:


$$\begin{array}{c} \frac{\partial \phi }{\partial t}+u_{j}\frac{\partial \phi }{\partial x_{j}}=\gamma \frac{\partial^{\text{2}}\phi }{\partial x_{i}\partial x_{j}}+s_{\phi } \end{array}$$ (3)

where $u_{i}$ is the instantaneous velocity component in the $x_{i}$ direction; p is the instantaneous pressure; ${\text{g}}_{\text{i}}$ is the force per fluid mass; ϕ is a scalar, which can represent temperature T or substance concentration C; $s_{\phi }\;$is the volume source term, such as the heat generated by chemical reactions or biological reactions; v is the kinematic viscosity coefficient of water; and γ is the ϕ molecular diffusion coefficient.

The k equation is as follows:


$$\begin{array}{c} \frac{\partial k}{\partial t}+\langle u_{j}\rangle \frac{\partial k}{\partial x_{j}}=\frac{\partial }{\partial x_{j}}\left(\frac{\nu_{t}}{\sigma_{k}}\frac{\partial k}{\partial x_{j}}\right)+P+G-\varepsilon \end{array}$$ (4)

The ϵ equation is as follows:


$$\begin{array}{c} \frac{\partial \varepsilon }{\partial t}+\langle u_{j}\rangle \frac{\partial \varepsilon }{\partial x_{j}}=\frac{\partial }{\partial x_{j}}\left(\frac{\nu_{t}}{\sigma_{\varepsilon }}\frac{\partial \varepsilon }{\partial x_{j}}\right)+C_{1\varepsilon }\frac{\varepsilon }{k}(P+C_{3\varepsilon }G)-C_{2\varepsilon }^{*}\frac{\varepsilon^{\text{2}}}{k} \end{array}$$ (5)

where p is the turbulent kinetic energy generation term caused by the average velocity gradient; G is the generation term of turbulent kinetic energy caused by buoyancy; $C_{\text{1}\varepsilon }$=1.42; $C_{\text{2}\varepsilon }$ =1.68; $\sigma_{\text{k}}$ =0.7194; and $\sigma_{\epsilon }$= 0.7194.

The expression of $C_{\text{2}\varepsilon }^{*}$ is as follows:


$$\begin{array}{c} C_{2\varepsilon }^{*}=C_{2\varepsilon }+\frac{C_{\mu }\eta^{\text{3}}(1-\eta /\eta_{\text{0}})}{1+\beta \eta^{\text{3}}} \end{array}$$ (6)

where $C_{\mu }$=0.0845; $\eta_{\text{0}}$=4.38; β=0.012; $\eta =\frac{Sk}{\varepsilon }$;$S={\left(\text{2}\left\langle S_{ij}S_{ij}\right\rangle \right)}^{\frac{\text{1}}{\text{2}}}$

### 2.2. Numerical methodology validation

To verify the reliability of the simulation in the software environment, the results were compared against physical model data [17] from the vertical-slot section of the fish passage facility at Dipan Hydropower Station in Qinghai Province.

#### 2.2.1. Verifying the model overview.

**Fig 1:**
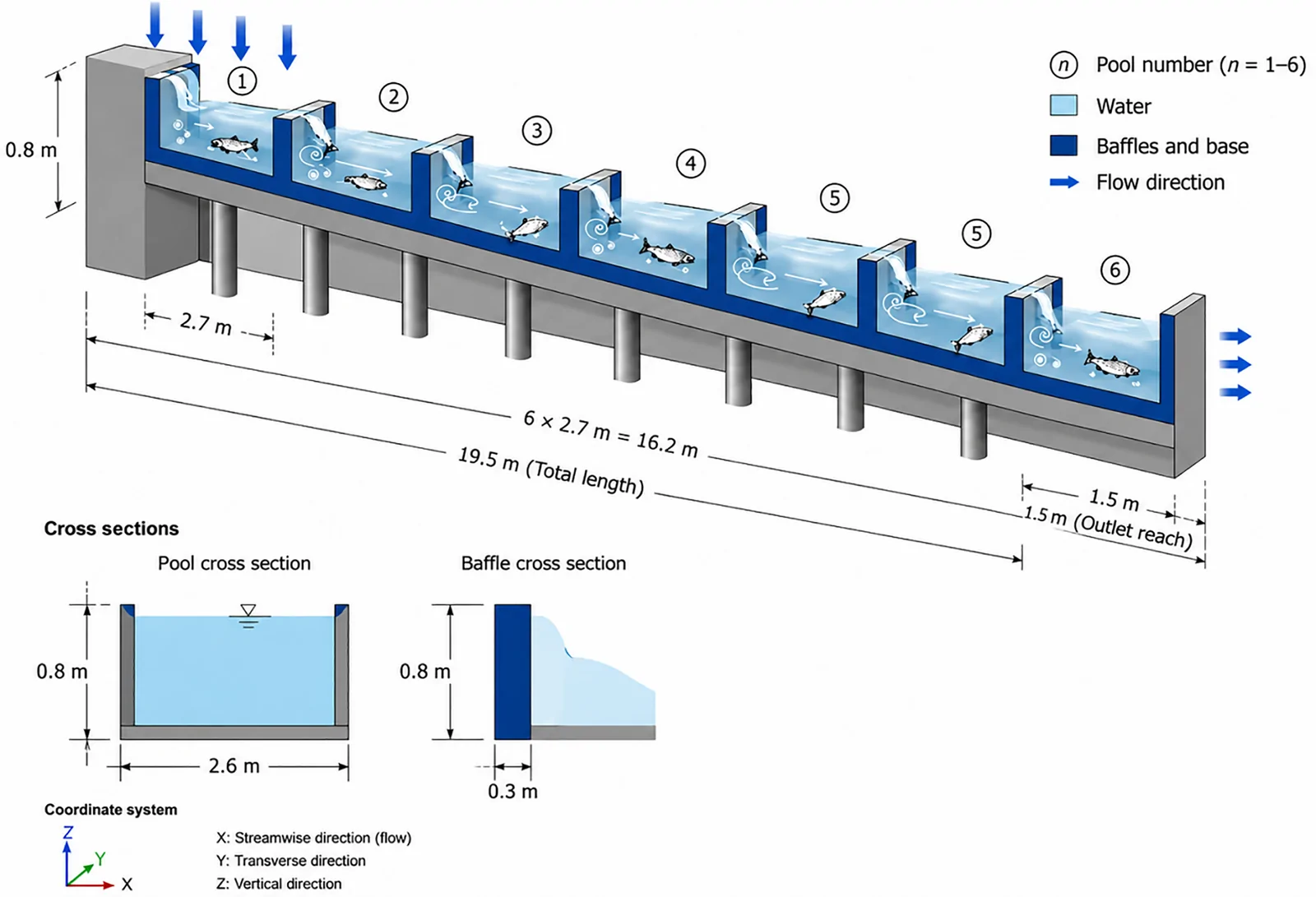
Model structure.

According to the gravity similarity criterion, the geometric scale of the physical model is 1:10, the length of the vertical joint is 18.4m, the length of the pool is 0.3m, the width of the pool is 0.2m, the height is 0.32m, the width of the vertical joint is 0.04m, the roughness is 0.018m, and the drop between the adjacent pool is 0.0056m. The actual fishway model’s bottom is covered in 2 cm-thick pebbles, and the whole is assembled with plexiglass. The vertical slit fishway section of the physical model’s conventional fishpond structure is precisely the same size as the three-dimensional model. Twenty pools and two resting pools are selected for simulation, and the total length of the calculation section of the model is 7.1m. A portion of the model was intercepted and is depicted in (Fig 1) to provide an adequate comprehension of the internal structure of the vertical slit fish route.

#### 2.2.2. Validation of numerical methodology.

To assess the reliability and accuracy of the selected numerical methods for simulating complex free-surface flows in fishway structures, a validation study was conducted. The validation leverages high-quality experimental data from a physical model test. While the geometry used for validation differs from the overflow weir fishway that is the primary focus of this study, the purpose here is to verify the solver’s capability in capturing key hydraulic phenomena common to many fishway types, such as free-surface deformation, turbulent flow structures, and the overall water level profile under controlled flow conditions [18]. This approach is commonly adopted in computational fluid dynamics studies when direct validation data for the specific geometry of interest is not readily available.

#### 2.2.3. Comparison and verification of simulation results.

The physical model test examined the average velocity at the vertical joint as well as the water depth before and after the baffle. After completing the simulation, the computed results were compared with the physical measurement, the calculated values were retrieved and compared with the measured values.

**Fig 2:**
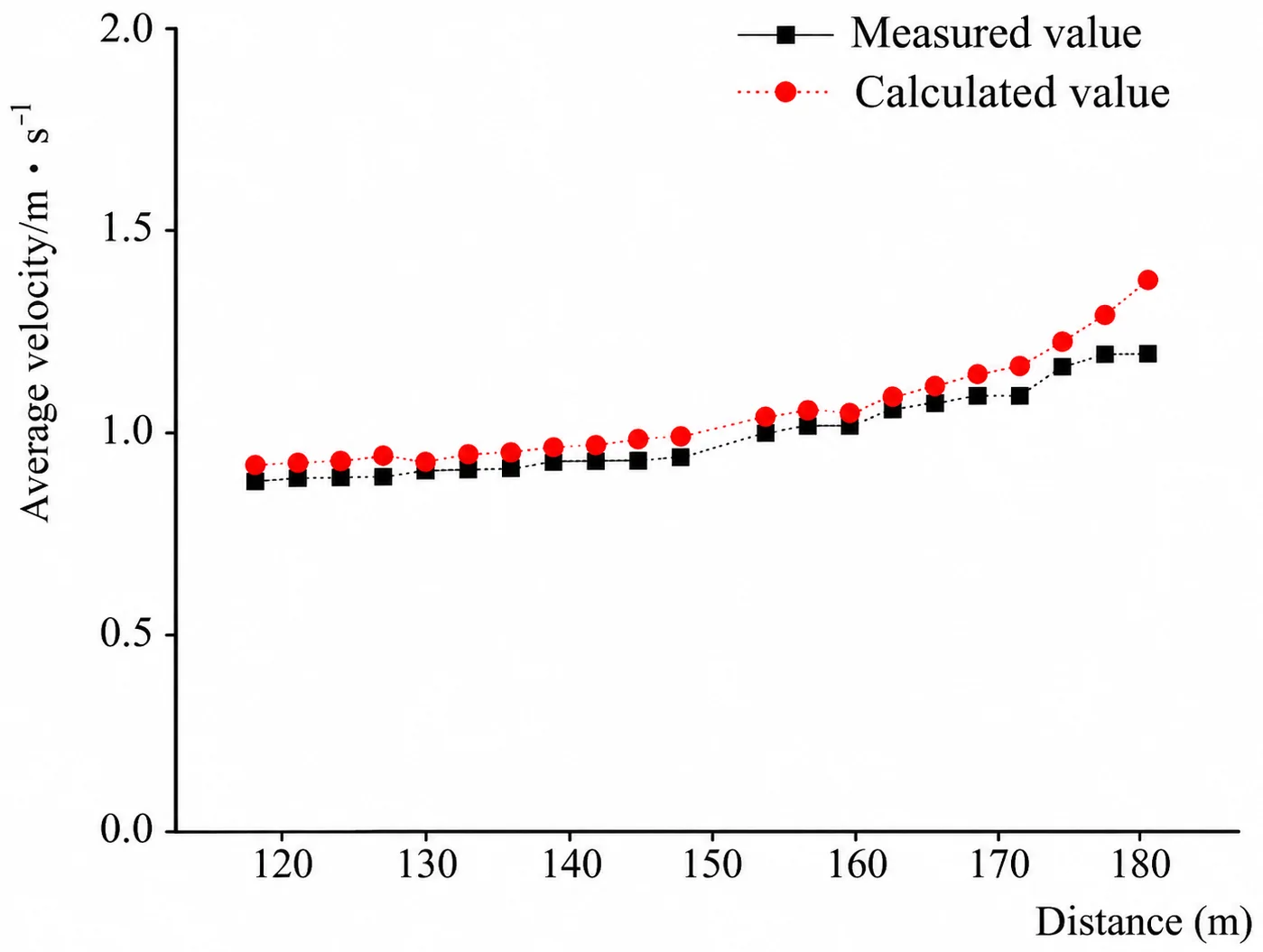
Verification of average velocity at vertical slot.

A comparison of (Fig 2) shows that the overall deviation between calculated and measured values is not significant. Specifically, the calculated average flow velocity at the vertical slot of the final pool is overestimated. This primarily stems from the fact that in the physical model, the vertical slot fishway terminates in a natural-flow channel. Consequently, the average flow velocity at the vertical slot within this pool is influenced by the operational conditions of the downstream pool. However, in the numerical simulation, this interaction is simplified by applying a pressure outlet lower boundary condition based on the controlled water level, which cannot fully replicate the actual flow dynamics. In the study of fishway hydraulic characteristics, a representative pool in the middle section will be selected for focused analysis. Therefore, the overestimation in the outlet section of the calculated fishway model does not affect the analysis of hydraulic characteristics.

**Fig 3:**
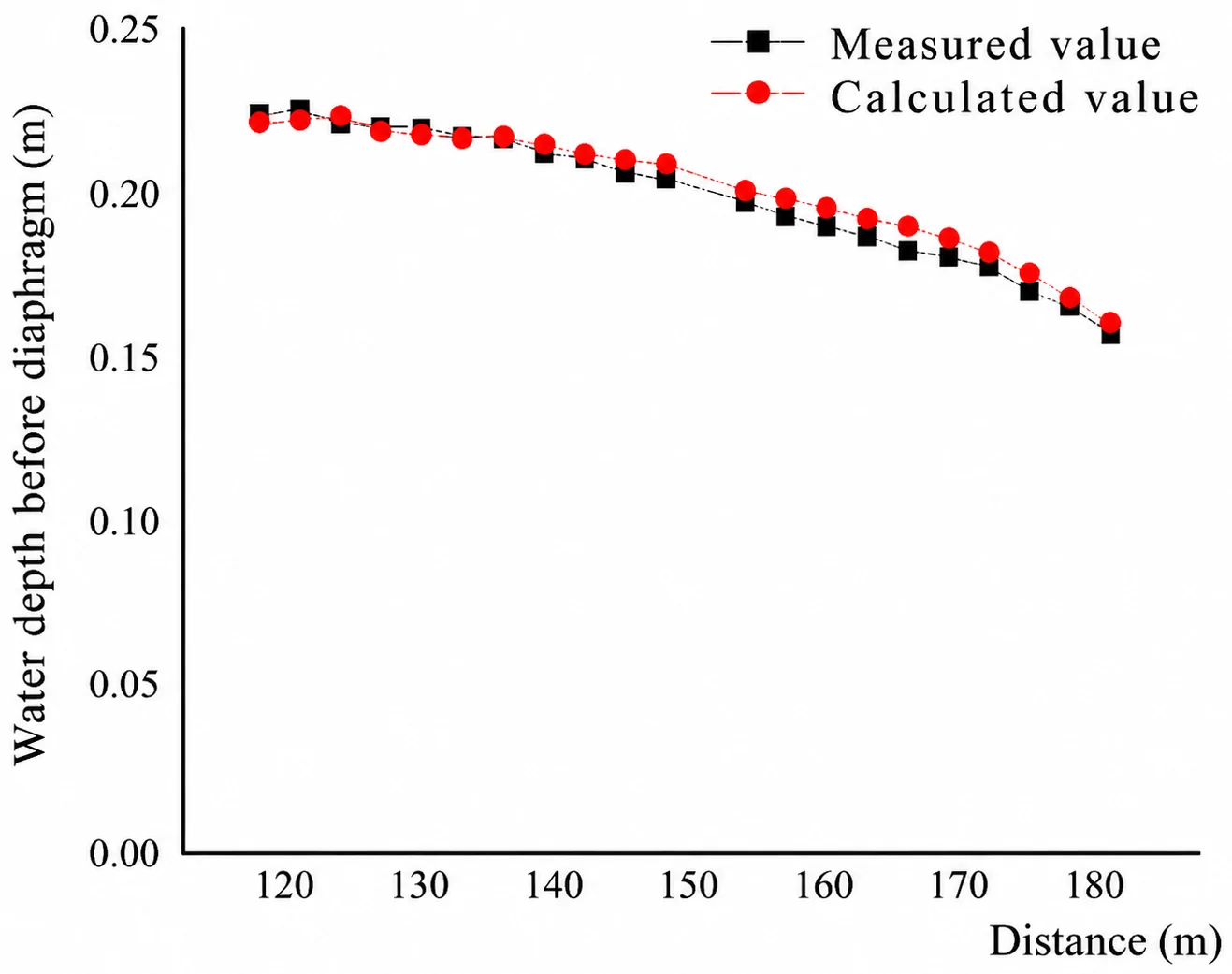
Verification of water depth before baffle.

**Fig 4:**
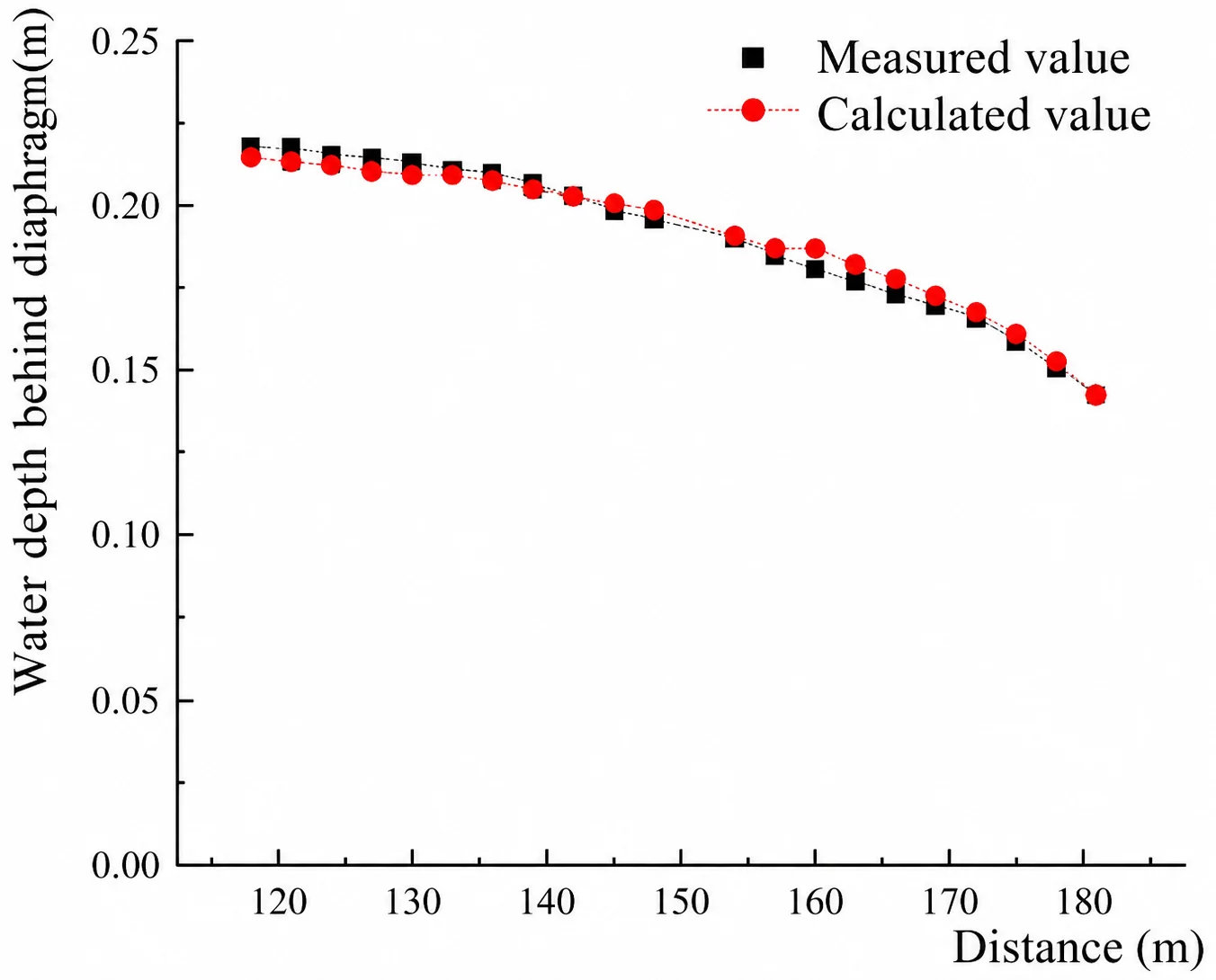
Verification of water depth behind the baffle.

The water depth before and after the baffle at the longitudinal central axis of the fishway was extracted and compared with the measured value. As can be seen from the comparison in (Fig 3) and (Fig 4), the calculated value fluctuates up and down from the measured value. The primary cause is that the fishway’s bottom is covered in 20 mm thick pebbles in the physical model, and because these pebbles are scattered and irregular, it is challenging to replicate the actual environment using simulation techniques. However, overall, there is a good fit between the measured and computed values.

The calculated values for average slot velocity, water depth before the baffle, and water depth behind the baffle show good agreement with the measured data, with relative errors of 4.8%, 1.42%, and 0.31%, respectively. These errors are within an acceptable range for engineering applications. This successful validation demonstrates that the employed numerical setup is capable of producing reliable results for fishway hydraulics involving free-surface flow, turbulence, and interaction with baffle structures.

It is important to acknowledge a limitation: The physical model used for validation is a vertical-slot fishway, which has a different flow mechanism compared to the overflow weir fishway studied in the main body of this paper. Therefore, this validation primarily confirms the competence of the numerical tools in a broader context of fishway flow simulation, rather than providing direct validation for the specific overflow weir configuration. The decision to proceed with the numerical investigation of the overflow weir fishway is based on the well-established application of the RNG k-ε and VOF models in simulating similar free-surface overflow problems in hydraulic engineering.The scarcity of publicly available experimental data specifically for overflow weir fishway models is a notable limitation of this study.

## 3. Scheme design

### 3.1. Model overview

The fishway model pool is 2.7m in length and 2.6 m in width. The baffle has a thickness of 0.3 meters and a vertical distance of 0.8 meters from the top to the bottom of the upper pool. The main geometric dimensions of the overflow-weir fishway model, including the number of pools, pool length and width, vertical drop, baffle thickness, and outlet-reach length, are summarised in Table 1.The length of the model is 19.5 m, with a total of six pools. Pools 1# to 6# are numbered in a downstream direction. The 1.5 m-long outlet segment is behind the last baffle. Since the vertical distance between the top of the baffle and the bottom of the next level pool directly affects the operating water depth of the overflow weir fishway, three different situations are set according to the experimental requirements: h = 0.925m, h = 0.95m and h = 0.975m.The bottom plates of the fishway pools are horizontal, and the water level difference between the upstream and downstream sections is created by a stepped arrangement. In this paper, the fishway longitudinal slope i is defined as the ratio of the vertical drop Δh between adjacent pools to the horizontal length of a single pool ${\text{L}}_{\text{pool}}$, given by the formula $i=\Delta h/L_{\text{pool}}$. Where i is the fishway longitudinal slope; Δh is the vertical drop between adjacent pools (m); and $L_{pool}$ is the horizontal length of a single pool, with ${\text{L}}_{\text{pool}}$=2.7 m.By adjusting the vertical drop between adjacent pools, the fishway slopes are set to i=0.038, i=0.046, and i=0.054, respectively.

**Table 1:** Model dimensions and parameters.

Number of pools	Pool length	Pool width	Vertical drop	Baffle thickness	Outlet reach length
6	2.7m	2.6m	0.8m	0.3m	1.5m

### 3.2. Grid division

**Fig 5:**
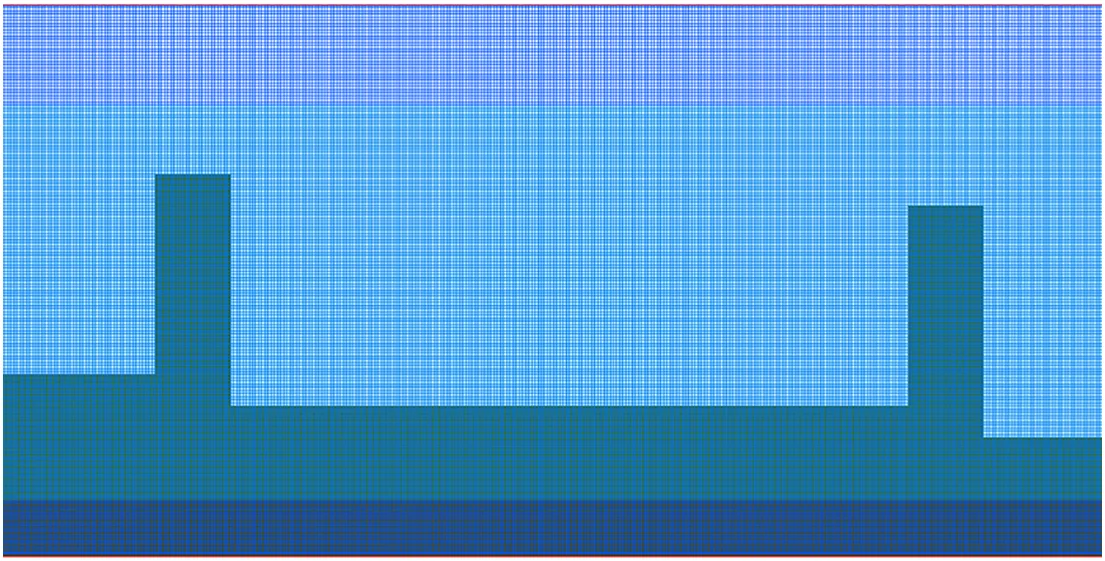
Schematic diagram of the mesh.

As seen in (Fig 5), uniform structured orthogonal grids were chosen for each model, and the calculation region was wrapped in a single grid block. The grid blocks were located between 0.1 and 3.1 meters in the y direction, between 0.0125 and 2.5 meters in the z direction, and between 0.0125 and 17.9875 meters in the x direction. 0.0125 m × 0.05 m × 0.0125 m is the mesh size, and there are 17,169,720 grids in total.

### 3.3. Boundary conditions and initial conditions

To ensure reproducibility, the simulation boundary conditions are precisely defined as follows:

Inlet boundary: A volume flow rate inlet boundary condition is applied. The flow rate is varied according to the specific working case 0.15m^3^/s, 0.20m^3^/s, and 0.25m^3^/s, and the corresponding upstream water level is specified.

Outlet boundary: A pressure outlet boundary condition is applied. To accurately simulate the overflow characteristics, the outlet boundary is set at the last baffle. A fixed fluid elevation is imposed at this boundary, which is determined by the crest elevation of the last baffle. This setup allows the water to overflow the baffle and exit the domain naturally under the specified downstream water level.

Top boundary: A specified pressure boundary condition is applied to track the free surface evolution of the water flow.

Walls: No-slip wall boundary conditions are applied. The standard wall function is used to resolve the near-wall turbulence, and the roughness height is set according to the physical model parameters.

Initial Conditions: To reduce computational time and ensure numerical stability, an initial hydrostatic water level is set within the pools, matching the inlet water level of the corresponding working case.

### 3.4. Working condition design

Three inlet discharge levels (0.15 m³/s, 0.20 m³/s, 0.25 m³/s) were set for the fishway, with the inlet water level adjusted accordingly. To explore the relationship between flow regime occurrence, inlet discharge, and fishway slope, two additional vertical drop levels (0.025 m and 0.05 m increase) were tested for each inlet discharge, corresponding to fishway slopes of 0.046 and 0.054, respectively. The detailed design of the 9 numerical simulation cases is listed in Table 2.

**Table 2:** Working case design of numerical simulation.

Operating condition number	Fishway type	Inlet discharge(m^3^/s)	Boundary condition water level in x direction (m)	Fishway model adjacent pool drop (m)	Fishway grade i
1	Overflow weir fishway	0.15	1.875	0.125	0.038
2	Overflow weir fishway	0.20	1.894	0.125	0.038
3	Overflow weir fishway	0.25	1.913	0.125	0.038
4	Overflow weir fishway	0.15	2.025	0.150	0.046
5	Overflow weir fishway	0.20	2.044	0.150	0.046
6	Overflow weir fishway	0.25	2.063	0.150	0.046
7	Overflow weir fishway	0.15	2.175	0.175	0.054
8	Overflow weir fishway	0.20	2.195	0.175	0.054
9	Overflow weir fishway	0.25	2.215	0.175	0.054

## 4. Results and analysis

### 4.1. Flow regime variation

**Fig 6:**
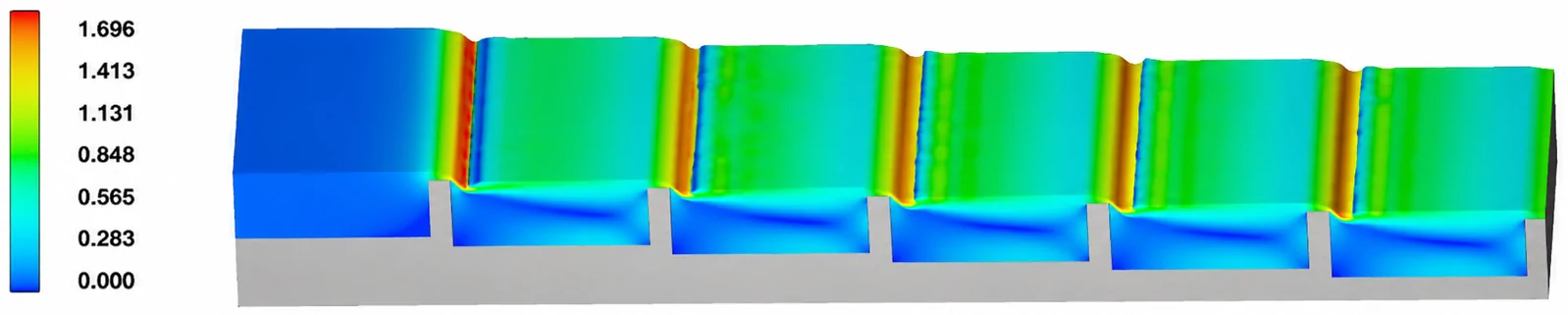
Streaming flow regime.

**Fig 7:**
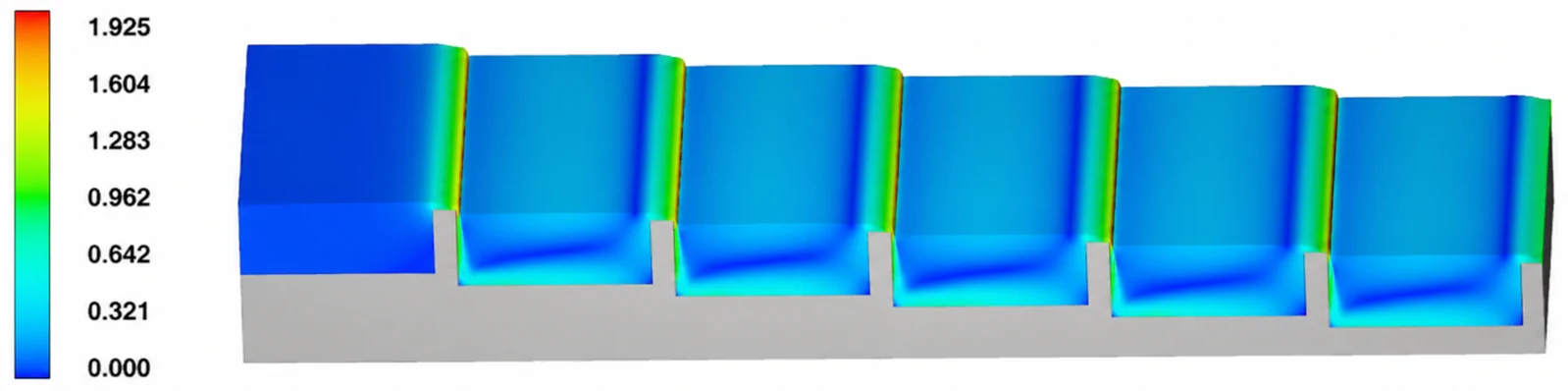
Plunging flow regime.

**Fig 8:**
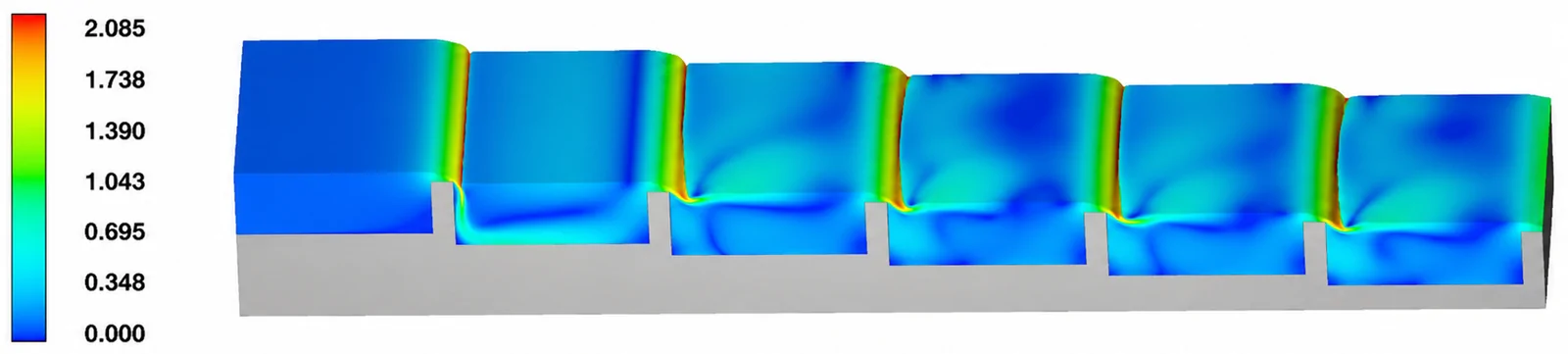
Transition flow regime.

The overflow weir fishway mainly overflows from the baffle to the next level by filling the upper-level pool with water. Due to the varied flow rates, the model of the same overflow weir fishway will display distinct flow patterns. As illustrated in (Fig 6), in the case of large flow, the main flow, also known as streaming flow, supports the main flow on the flow’s surface. (Fig 7) illustrates what is known as plunging flow, which occurs when the mainstream dips after passing through the baffle at a low flow rate. Between large flow and small flow, the main flow will swing back and forth between the surface and the bottom plate of the pool. As illustrated in (Fig 8), this crucial state is known as transition flow.

Streaming flow is a phenomenon that typically occurs in huge flows. The partial velocity of the velocity in the x direction is higher because of the high flow rate. Following its passage through the baffle, the water travels downstream, directly impacted by the partial velocity in the x direction, rather than plunging beneath the force of gravity. The baffle, which tops the mainstream and floats on the water’s surface, creates a sizable recirculation zone below the mainstream. In this situation, the velocity on the pool surface is larger than the velocity of water flow in the pool return region.

Plunging flow is typically a phenomenon that happens in small flows. After passing through the baffle plate, the main water flow will plunge under the force of gravity due to the low flow velocity. After passing through the bottom plate, the water flows downstream until it reaches the next baffle plate, where it rises and traverses the baffle plate of the next level of the pool. The mainstream descends to the bottom in the middle of the pool, and the mainstream is compressed by the reflux area, which is noticeably distinct from the streaming flow.

**Fig 9:**
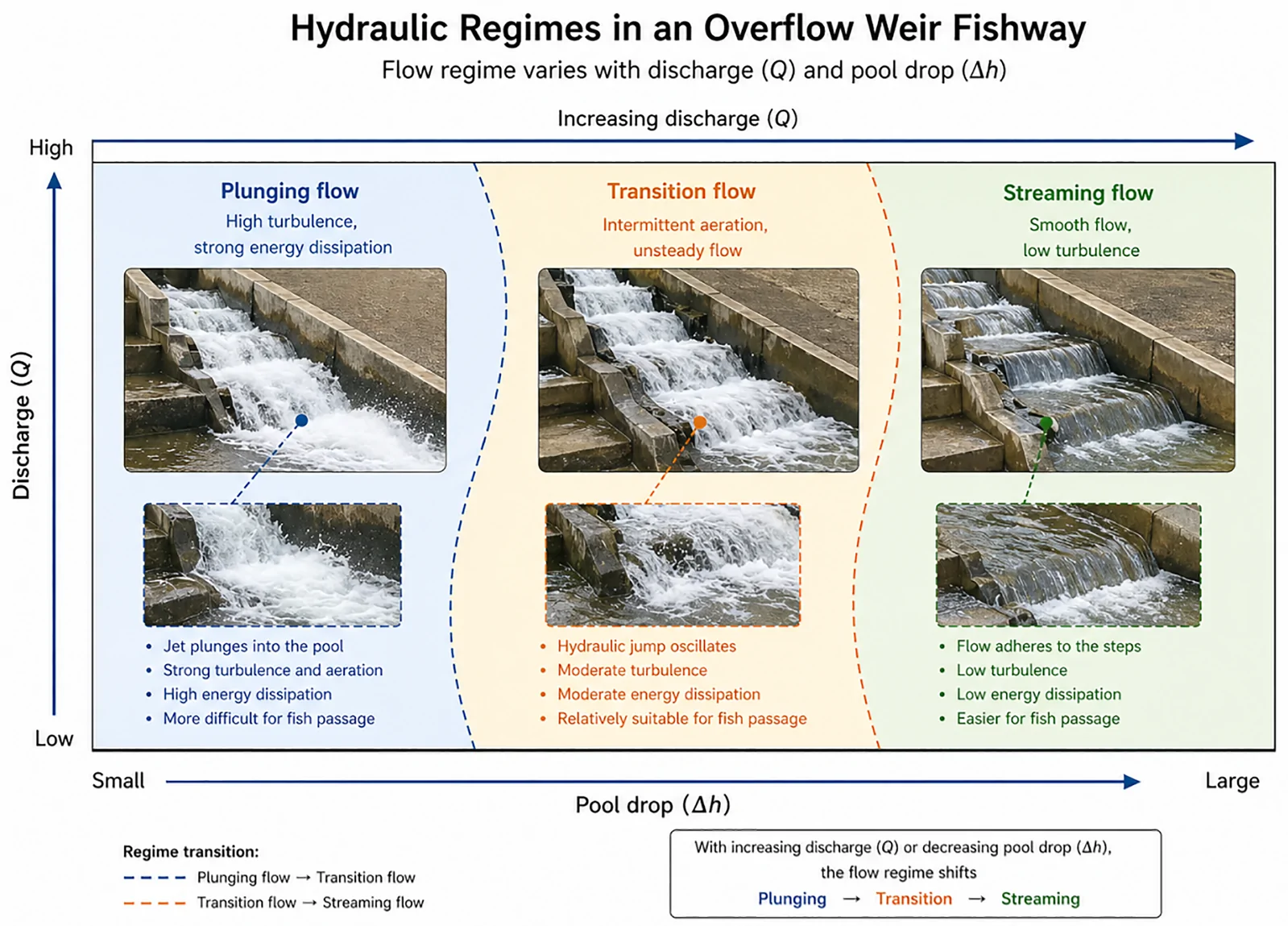
Hydraulic Regimes in an Overflow Weir Fishway.

Between streaming flow and plunging flow, there is a key stage known as the transition flow phenomenon. The primary flow does not vary up and down simultaneously in the y direction of the pool; Instead, it swings between streaming flow and plunging flow. It can be thought of as the process by which the flow pattern changes from falling to sliding as a result of an increase in flow rate. The flow rate associated with the transition flow phenomenon is an interval rather than a critical value, and it varies with the drop between adjacent pools and the inlet flow.The overall relationships among inlet discharge, inter-pool vertical drop, and three distinct hydraulic regimes are summarized in (Fig 9), which illustrates the regime transition boundaries and qualitatively compares turbulence characteristics and fish-passage suitability for plunging flow, transition flow, and streaming flow.Nine working circumstances were determined for comparison in order to explore the change rule of water flow from plunging flow to transition flow and finally to streaming flow. Table 3 displays the detailed calculation findings.

**Table 3:** Calculation results of the flow regime under different cases.

Working condition	The drop between adjacent pools (m)	Slope i	Inlet flow (m^3^/s)	Flow pattern
Condition 1	0.125	0.038	0.15	Transition flow
Condition 2	0.125	0.038	0.20	Streaming flow
Condition 3	0.125	0.038	0.25	Streaming flow
Condition 4	0.150	0.046	0.15	Plunging flow
Condition 5	0.150	0.046	0.20	Transition flow
Condition 6	0.150	0.046	0.25	Streaming flow
Condition 7	0.175	0.054	0.15	Plunging flow
Condition 8	0.175	0.054	0.20	Plunging flow
Condition 9	0.175	0.054	0.25	Transition flow

The computation results show that there is a certain rule between the input flow, the drop of the neighboring pool, and the water flow pattern in the pool. The transition flow mode appears when the inlet flow rate is 0.15 m^3^/s, the streaming flow mode appears when the inlet flow rate is 0.20 m^3^/s, and the streaming flow mode appears when the inlet flow rate is 0.25 m^3^/s,when the drop of the adjacent pool is 0.125 m. In other words, when the inflow discharge exceeds 0.20 m³/s, the flow regime becomes streaming flow. The water flow pattern was plunging when the inflow flow rate was 0.15 m^3^/s. The water flow pattern changed to a transition flow pattern when the inflow flow rate was 0.2 m^3^/s. The water flow pattern displayed a streaming pattern at a flow rate of 0.25 m^3^/s. If the fishway slope is 0.054, the inlet flow is 0.15 m^3^/s and 0.2 m^3^/s, and the transition flow occurs when the flow is 0.25 m^3^/s, then the drop between adjacent pools is further deepened by 0.025 m. As the inlet flow changes from tiny to large, it is evident that plunging flow, transition flow, and streaming flow exist in the same order. The more probable it is for plunging flow to occur with the same drop between neighboring pool in a fishway, and the more probable it is for streaming flow to occur with a larger inlet flow; More drop between adjacent pools makes it simpler to have a plunging flow pattern at the same intake flow rate, while less drop between neighboring pools makes it easier to have a streaming flow pattern.

### 4.2. Velocity distribution

The velocity distribution of different flow patterns is quite different. Conditions 4 and 6 are selected as two typical conditions of plunging flow and streaming flow for analysis. The research range was chosen to include pool 4# and the front baffle (x = 8.09375 ~ 11.09375m) in order to comprehend the velocity distribution of the most stable water flow structure.

**Fig 10:**
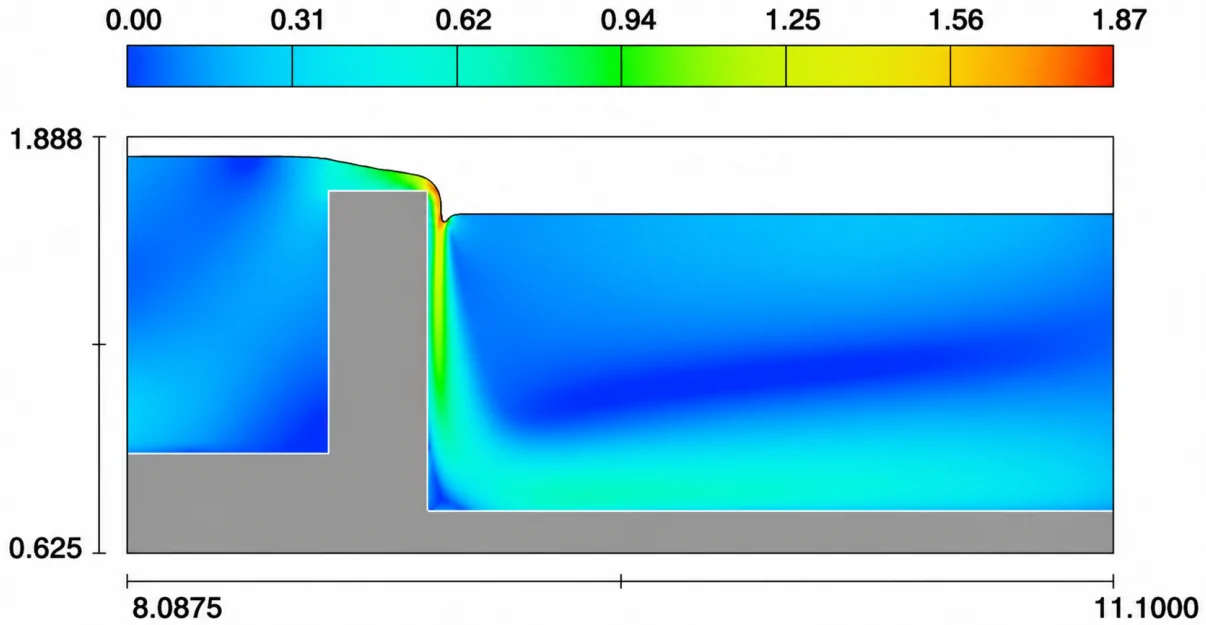
Case 4 Velocity distribution diagram.

**Fig 11:**
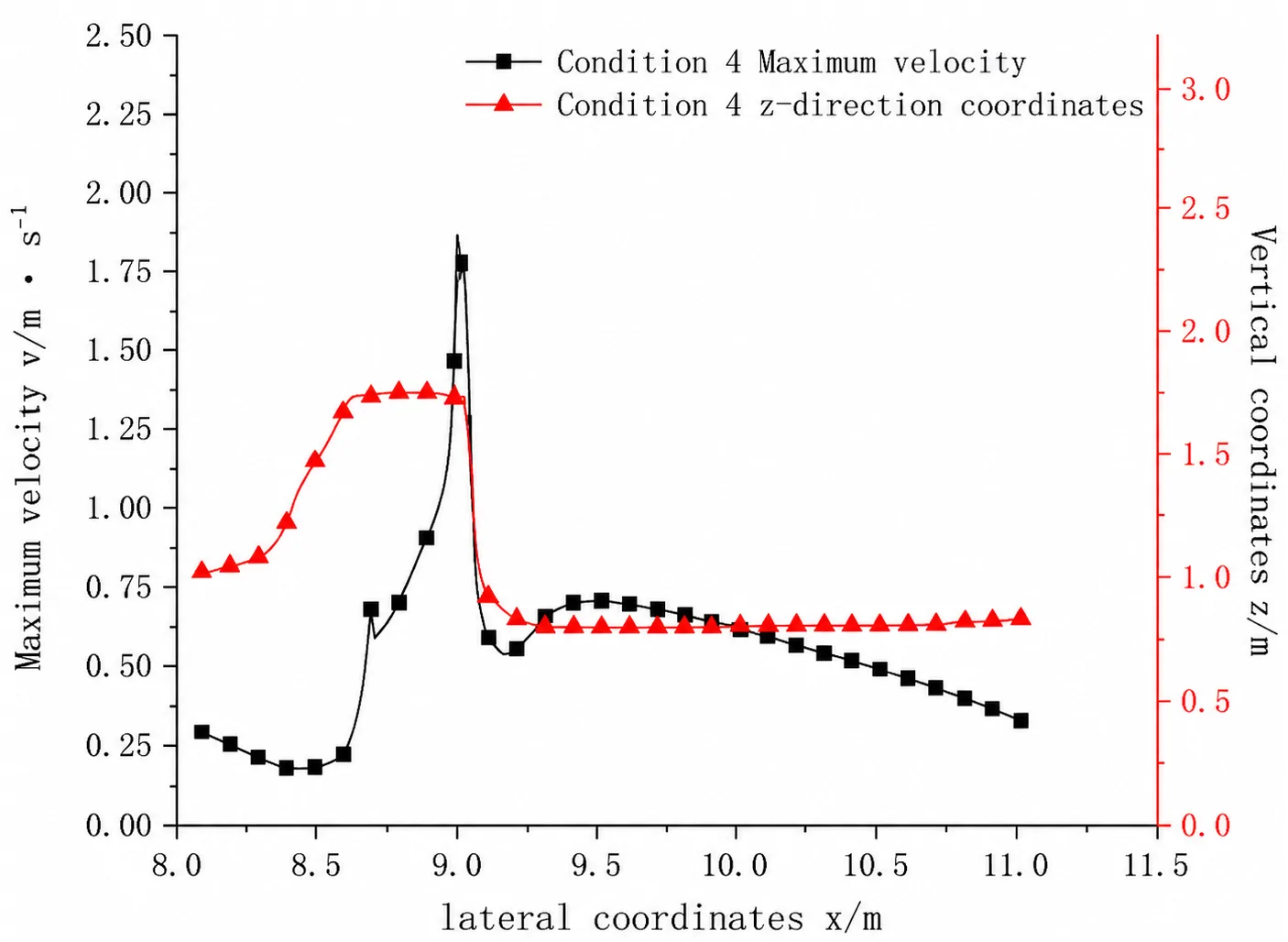
Case 4 Maximum velocity and position along the way.

**Fig 12:**
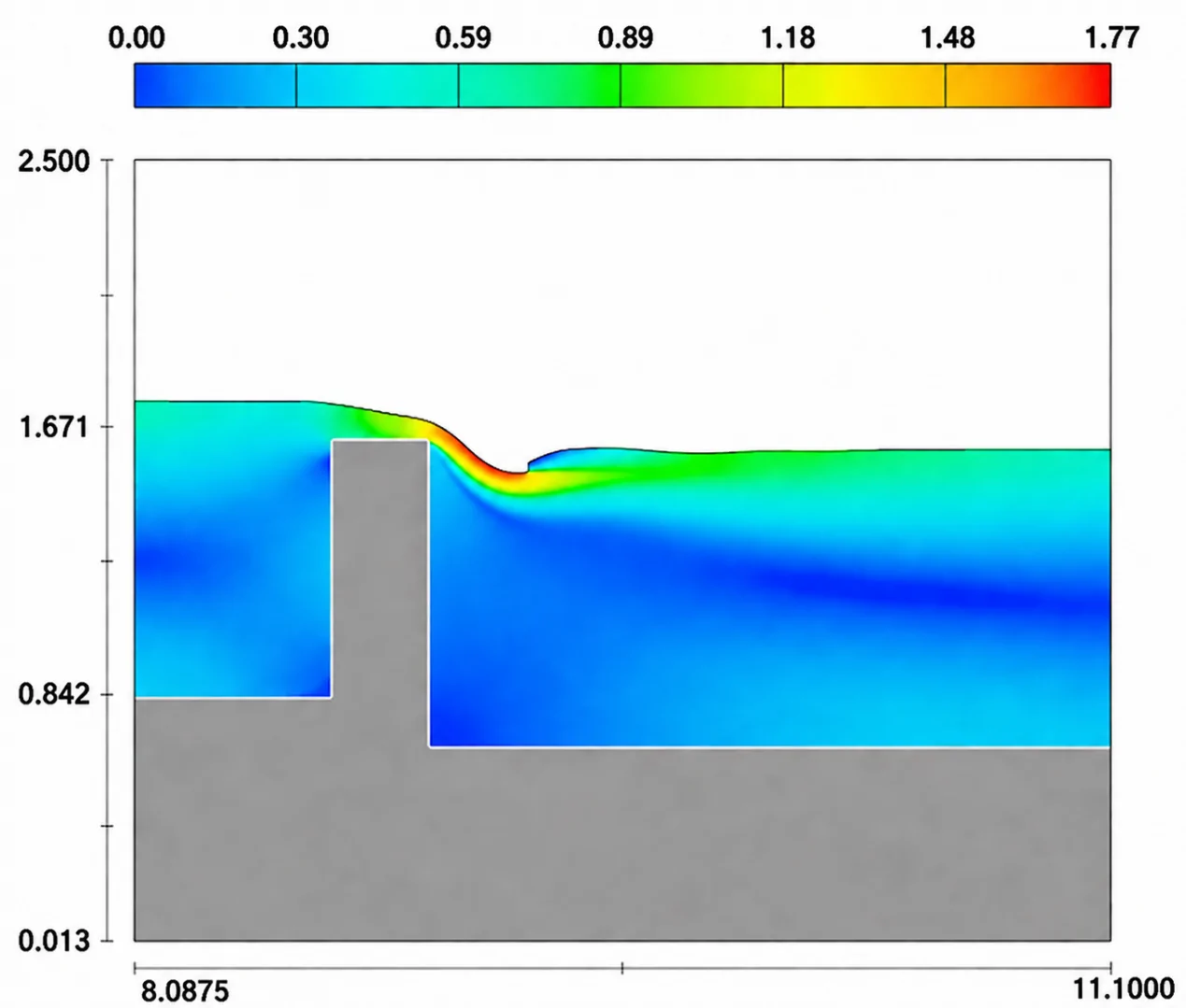
Case 6 Velocity distribution diagram.

**Fig 13:**
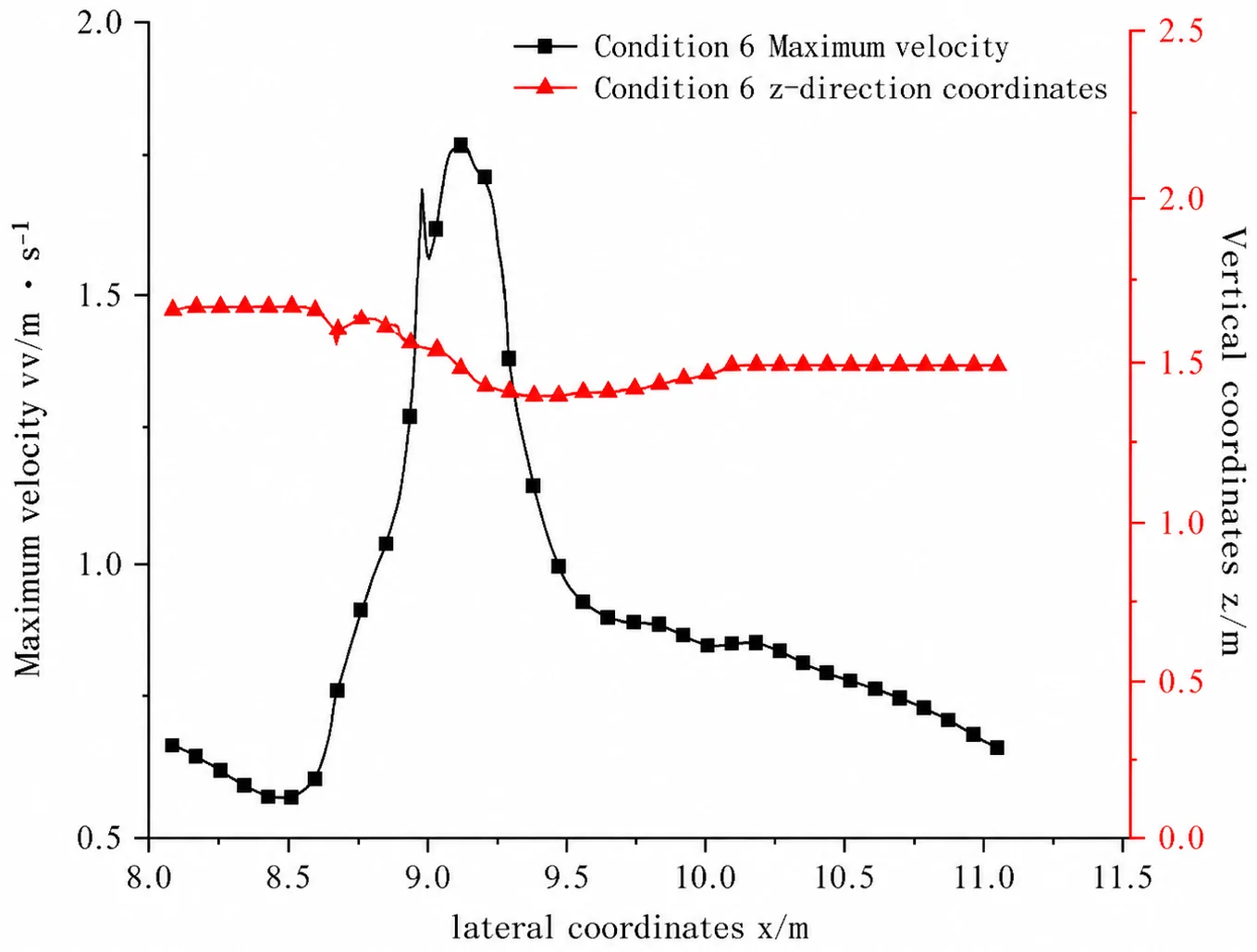
Case 6 Maximum velocity and position along the way.

The velocity distribution diagram in the x-z direction is displayed in (Fig 10) for working condition 4, y = 1.625m (the middle location of the fishway pool), assuming that the drop of the neighboring pool is 0.15m and the inlet flow is 0.15m3/s. It can be seen intuitively that the flow mode in working condition 4 belongs to plunging flow. By comparing the distribution of the maximum velocity along the path and its position in the z direction, as shown in (Fig 11), it can be seen that the maximum velocity of the whole pool occurs at the position where the water flow just flows through the position of the baffle (x = 9.00625), and the maximum velocity reaches 1.86m/s. The maximum flow velocity of the mainstream decays from x = 8.09375 m, and increases sharply after encountering the baffle. This sharp acceleration occurs as the flow passes over the weir crest, where gravitational potential energy is converted to kinetic energy, consistent with the well-documented physics of flow over a spillway. The velocity then decays rapidly after crossing the baffle.The maximum flow velocity of the mainstream decays quickly in the x direction, although the direction of the mainstream declines vertically at x = 9.06875 ~ 9.13125m. The velocity drops from 1.86m/s to 0.51m/s just past the threshold. Following its meeting with the next level of the pool of the baffle, which rises, the mainstream changes direction and continues to flow along the x direction. The area of the mainstream along the x direction is continuously growing, and the mainstream of the maximum flow rate along the x direction is gradually reduced. The mainstream by the upper part of the reflux area extrusion over the water cross-section reduces the flow rate and keeps getting bigger. The maximum flow velocity of the mainstream, which steadily decreases in all directions around it, is the center of the magnitude of the water flow velocity in the other sections of the pool. The surface of the pool flow, at x = 9.04 ~ 9.08 meters, is primarily where the higher flow velocity is concentrated, with a range of 1.00 ~ 1.86 meters per second. The main flow zone’s vast majority has flow velocities that are less than 1 m/s.

(Fig 12) depicts the velocity distribution diagram in the x-z direction under the parameters of 0.15m drop between neighboring pools and 0.25m3/s inlet flow, working condition 6, y = 1.625m (the middle location of the fishway pool). The streaming flow pattern and the plunging flow pattern have the same distribution in the y direction. (Fig 12) can represent the typical flow pattern of streaming flow. The distribution trend of velocity size is mainly studied. As can be seen from (Fig 13), the main flow of streaming flow floats on the surface of the pool, so the position of the maximum velocity of the main flow along the path does not change much. The main flow tends to descend slightly after passing the baffle before floating on the pool surface. The point at which the water goes through the baffle and appears in the underflow state (x = 9.15625m) is where the highest velocity occurs. The greatest velocity reaches 1.77 m/s when compared to the place where the maximum velocity occurs in the plunging flow state. Prior to the baffle, the main flow’s maximum velocity variation amplitude resembles that of the plunging flow; Nevertheless, in terms of velocity, the streaming flow is clearly higher than the plunging flow. When the water flow approaches the baffle and passes through it, its velocity increases gradually until it reaches its peak. After that, the attenuation range of the maximum velocity gradually reduces as the main flow steadily declines due to the mixing of the upper and lower reflux zones until the main flow reaches the water’s surface. The pool water surface, located at x = 8.88 ~ 9.21 meters, is primarily home to the high-velocity area, which has a velocity range of 1.00 ~ 1.77 m/s. In contrast, the major flow area has a velocity range of less than 1m/s.

The nine working conditions were split up into three groups: the input flow was set at 0.15 m^3^/s, 0.20 m^3^/s, and 0.25 m^3^/s, respectively, while the drop difference between neighboring pools was 0.125 m, 0.150 m, and 0.175 m. It is evident from the comparison that there are certain variations in the flow rates of various working conditions. Table 4 displays the velocity computation results for each working situation. In general, the flow velocity in the mainstream area increases with the increase of the drop between adjacent pools, and for the same flow pattern, the flow velocity in the mainstream area will increase with the deepening of the fishway pool and the increase of the inlet flow. In the three working conditions of the same fishway model, the maximum velocity always appears in the working condition corresponding to the transition flow mode. The main reason is that the flow mode of the transition flow is unstable, the main flow area always swings back and forth in the pool, the flow direction in the pool is chaotic, and the maximum velocity is easily achieved. Therefore, in the construction of the overflow weir fishway project, the occurrence of the transition flow mode should be avoided as far as possible.

**Table 4:** Calculation results of the flow rate under different cases.

Condition	The drop between adjacent pools (m)	Inlet flow (m^3^/s)	Flow pattern	Maximum flow rate of mainstream (m/s)	Flow velocity range in the mainstream zone (m/s)
Condition 1	0.125	0.15	Transition flow	1.72	——
Condition 2	0.125	0.20	Streaming flow	1.61	0.54 ~ 1.61
Condition 3	0.125	0.25	Streaming flow	1.67	0.56 ~ 1.67
Condition 4	0.150	0.15	Plunging flow	1.86	0.62 ~ 1.86
Condition 5	0.150	0.20	Transition flow	1.87	——
Condition 6	0.150	0.25	Streaming flow	1.77	0.59 ~ 1.77
Condition 7	0.175	0.15	Plunging flow	1.87	0.59 ~ 1.87
Condition 8	0.175	0.20	Plunging flow	1.89	0.64 ~ 1.89
Condition 9	0.175	0.25	Transition flow	1.95	——

**Fig 14:**
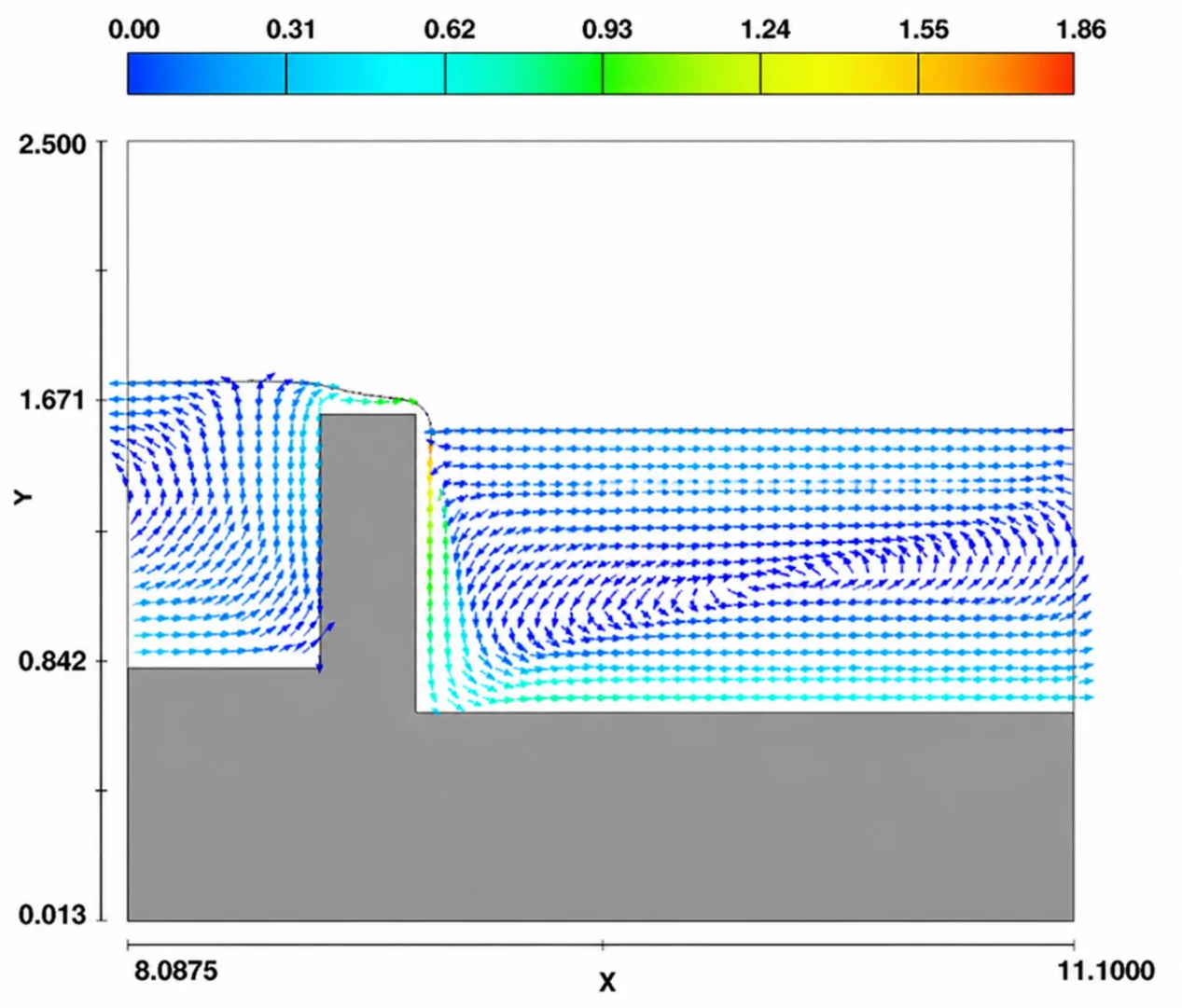
Case 4 Velocity vector diagram.

**Fig 15:**
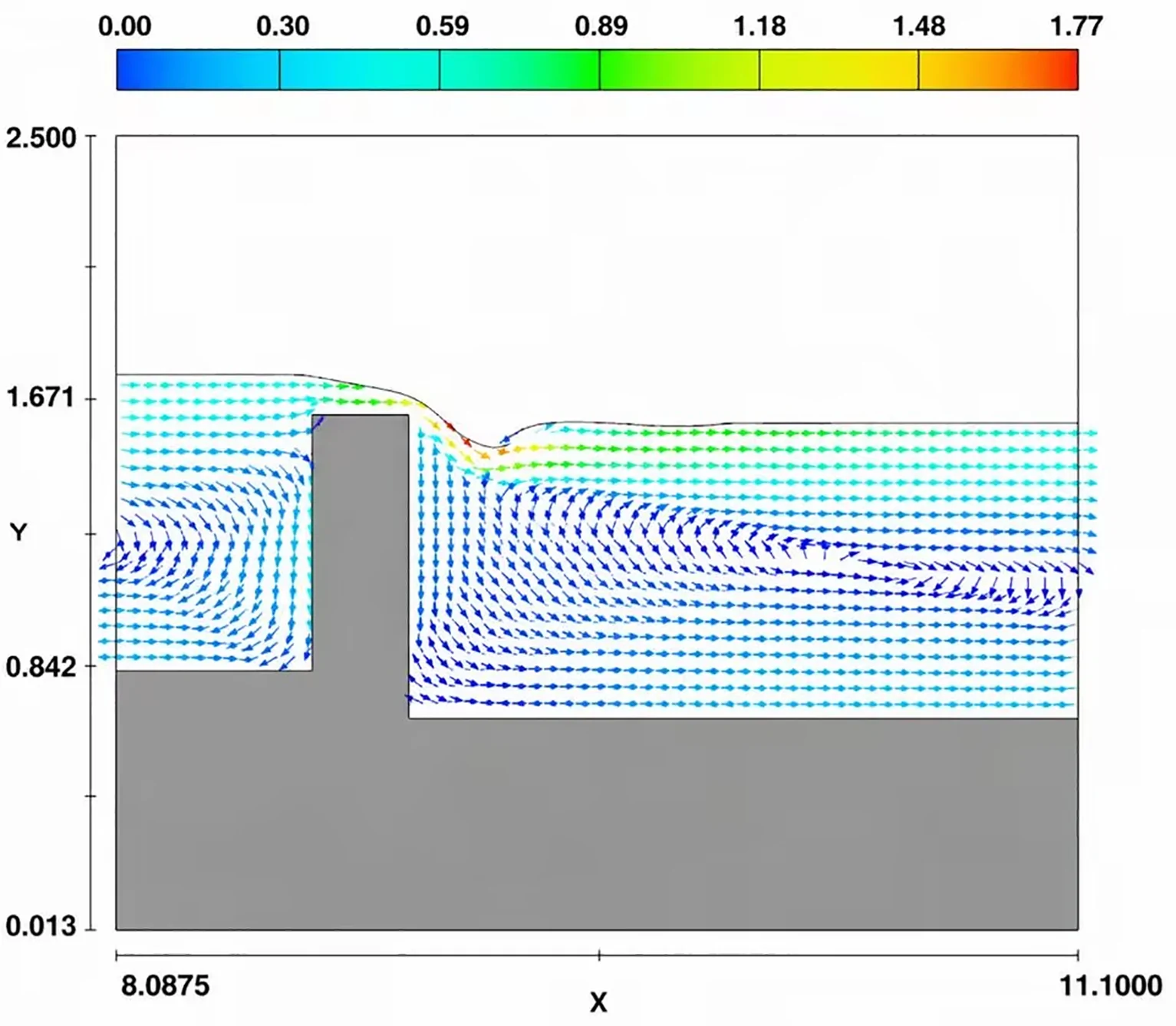
Case 6 Velocity vector diagram.

Whether it is a plunging flow or a streaming flow, the water flow is steady and predictable while the overflow weir fishway is in operation. The plunging flow in working condition 4 and the streaming flow in working condition 6 are used as examples, shown in (Fig 14 and 15). It is clear where the return flow ends and the main flow begins. The drop pool has only one vortex, which is placed above the mainstream, and the water flow line in the pool is stable. In the pool of streaming flow, there are two vortices. The enormous vortex is located in the center of the pool and at the bottom of the mainstream. The primary flow passes past the baffle, causing the little vortex to emerge as a weak water jump. The ratio of the water depth after the leap to the water depth before the jump is 3.32, while the Froude number prior to the jump is 1.78. Energy dissipation is greatly aided by the intense mixing and collisions among the fluid particles in this area.

The primary distinction between the plunging flow and streaming flow patterns in the fish passage is that the center of the vortex in the middle of the pool serves as the demarcation line, with the plunging flow pattern situated above the center of the vortex and the direction of the water flow in alignment with the migratory fish swimming in that direction, and the center of the vortex below the river channel’s water flow direction, in opposition to the fish swimming direction; The direction of the water flow, with the center of the vortex below the center of the swimming fish, presents a streaming flow pattern of the pool above the river channel’s center is the swimming direction of fish. This directly relates to the depth at which fish swim in the fishway. In many fish species, adult individuals generally exhibit active rheotaxis by swimming against the current and adjusting their orientation with flow changes. By contrast, juvenile fish often show more passive flow-following behavior along the mainstream direction. Such behavioral differences are species-specific and should be considered a general trend rather than a universal rule.

### 4.3. Turbulent kinetic energy and turbulent dissipation rate

**Fig 16:**
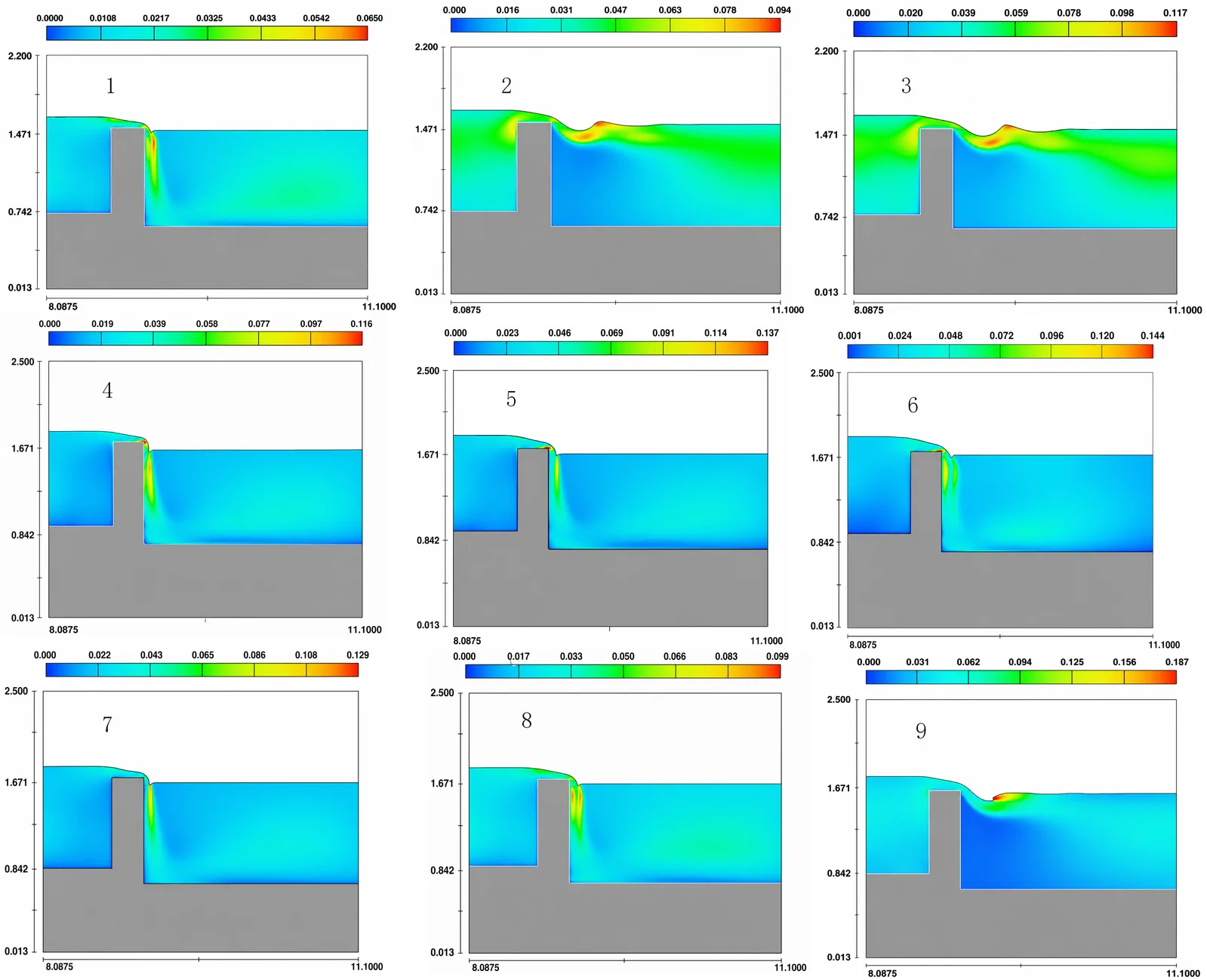
Turbulent kinetic energy diagram under different cases.

The distribution of flow patterns in overflow weir fishways and the turbulent kinetic energy distribution are somewhat correlated. The place where the water flow just passes through the baffle and the water tongue appears is where the maximum turbulent kinetic energy in the pool with a fall-off flow state occurs, as illustrated in (Fig 16). The pool with streaming flow has the highest turbulent kinetic energy at the end of the leap segment. However, as the flow pattern oscillates, so does the location of the maximum turbulent energy in the pool with the transition flow pattern. When compared to (4), (7), and (8) in (Fig 16), the turbulent energy in the pool generally rises as the flow rate increases; However, the turbulent energy in the large reflux area is relatively small, growing only slightly at the main flow tongue behind the baffle, and decreases in a circular pattern with water depth. pool flow is in a streaming flow state, and the main flow floats on the pool surface as compared to (2), (3), and (6) in (Fig 16). Furthermore, the total pool surface turbulence energy is relatively high; The area with high turbulence energy increases with flow, and the turbulence energy increases with flow closer to the baffle. Through a comparison with (4) and (7), (3) and (6) in (Fig 16), it is discovered that, for the same flow mode, raising the drop difference between neighboring pools at the same flow rate will partially reduce the turbulent kinetic energy overall, particularly in the middle of the pool where the reflux area is located, but it will not significantly affect the baffle.

**Fig 17:**
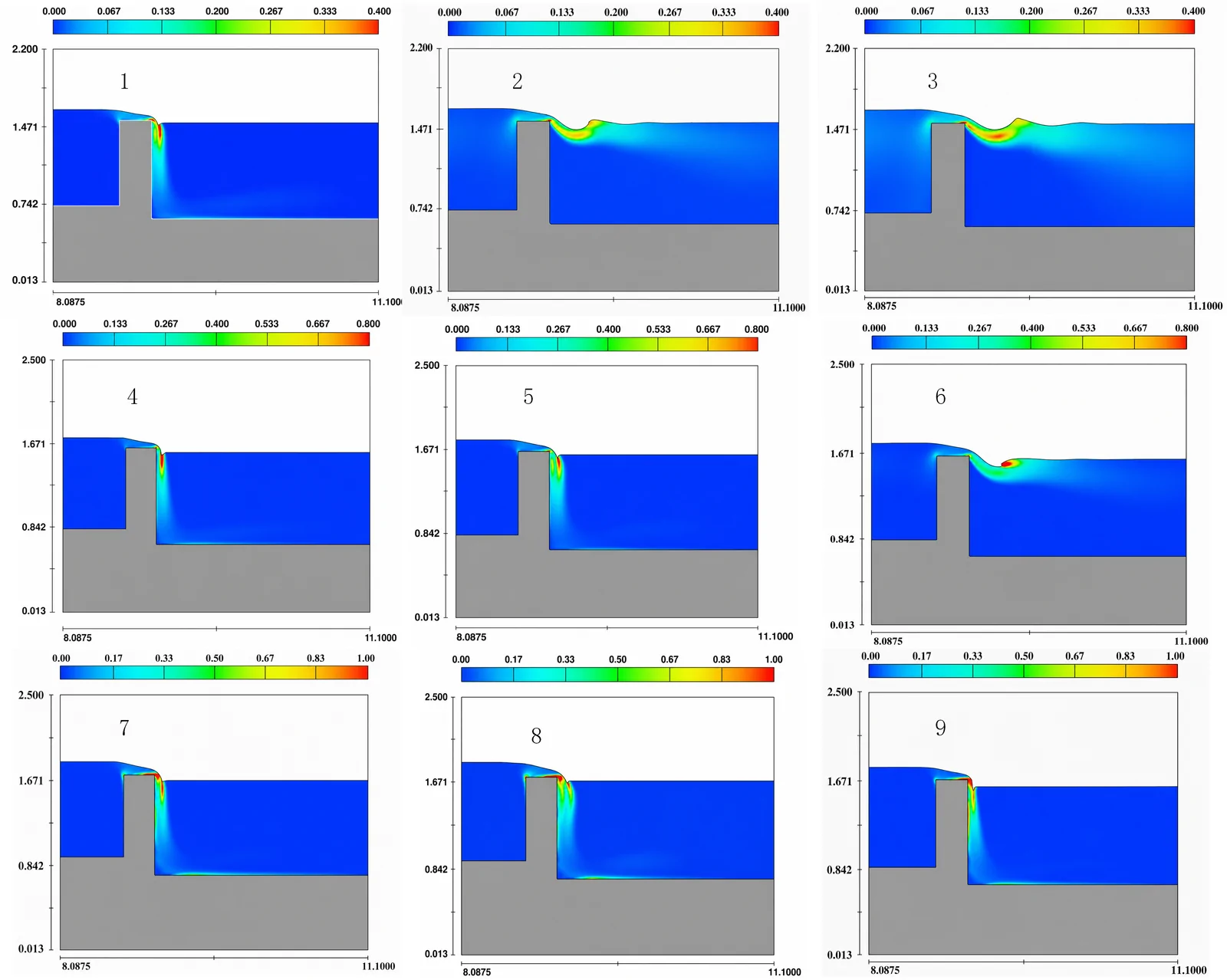
Turbulent dissipation diagram under different cases.

Furthermore, in regions with relatively high turbulent kinetic energy, the turbulent dissipation rate is also relatively high. (Fig 17) displays the turbulent dissipation rate distribution under various situations. When the flow passes through the baffle in a streaming flow pattern, the areas with high turbulent dissipation rates are mostly found in the turbulent kinetic energy area and the floating water surface, whereas the return flow area in the middle of the pool has a comparatively low turbulent dissipation rate. In the case of a plunging flow pattern, the turbulent dissipation rate is comparatively low in the center of the pool and on its surface, and it is mostly concentrated in the upper portion of the baffle plate, near its rear side, and the front half of the pool bottom plate. For transitional flow mode, the specific flow pattern formed by the main current in individual pool compartments must be assessed. Different scenarios follow distinct dissipation rate distributions characteristic of either streaming flow or plunging flow. After passing over the baffle, due to intense flow mixing and turbulence, the velocity gradient of the water becomes notably steeper, resulting in a relatively high dissipation rate in this region. This reflects the energy dissipation function of the fishway within the river channel.

## 5. Discussion

This study employs numerical simulation to investigate the hydraulic characteristics of an overflow weir fishway, aiming to clarify its behavior, limitations, and applications. The core function of any fishway is to facilitate upstream migration through energy dissipation, a process that varies fundamentally depending on the fishway type. By critically comparing the flow patterns, velocity distributions, and turbulent kinetic energy (TKE) found in this study with those of vertical-slot, Denil, and combined fishways, we can better understand the specific design implications and limitations of overflow weirs.

### 5.1. Flow patterns and energy dissipation mechanisms

The overflow weir fishway dissipates energy in a manner akin to a tiered spillway. Similar to the flow regime discrimination on stepped overflow surfaces analyzed by Han [19] and [20], our results confirm that the overflow weir fishway exhibits three distinct flow patterns: plunging flow, transition flow, and streaming flow, as originally proposed by Rajaratnam et al. [21]. However, unlike stepped spillways where aeration plays a key role [22,23], fishway energy dissipation is primarily governed by local head losses. In vertical-slot and Denil fishways, energy is dissipated through submerged jets creating intensive local mixing. In contrast, the overflow weir achieves this by vertically raising the water surface to form a free overflow. Our findings show that the presence of these three flow modes is highly sensitive to pool depth and inlet flow. By designing nine operational circumstances, we accurately grasped the critical inlet flow value corresponding to the transition flow mode under the same adjacent pool drop, complementing the theoretical formula validated by Masayuki Fujihara [24]. For fish passage design, transition flow represents a critical threshold; exceeding it shifts the regime to streaming flow, which alters the spatial location of the mainstream and subsequently affects fish migration trajectories, as migrating fish predominantly select the interface between the main flow zone and the recirculation zone [25–28].

### 5.2. Velocity Distribution: Overflow Weir vs. Vertical-Slot and Denil Fishways

The velocity range and maximum velocity directly dictate whether fish can migrate effectively, and guaranteeing variable flow conditions is crucial for accommodating different species and sizes to achieve biodiversity conservation [29,30]. Our study computes the flow velocity range in the primary flow area, revealing a distinct spatial distribution compared to other fishway types. In vertical-slot fishways, the mainstream often exhibits a high-velocity jet attached to the baffles, with slot velocities typically ranging from 0.76 to 0.89 m/s, while recirculation zones drop to 0.09–0.25 m/s [31]. Denil fishways, while simple to construct, produce highly turbulent flows suitable only for strong swimmers. In contrast, the overflow weir fishway generates a concentrated surface jet over the crest. Research on combined fishways shows that overflow weir crest velocities can reach 0.90–1.12 m/s, and dedicated guide weirs can produce even higher barrier velocities of 1.5–2.8 m/s to block and direct fish [32]. Unlike the 3D velocity attenuation in vertical slots explored by [33], our overflow weir model shows that if the maximum pool velocity exceeds the fishes’ burst speed, upstream movement is blocked. Conversely, as noted by [34–37], if the velocity in the pool’s main body is too low, fish easily become lost. Therefore, the vector-graph analysis of flow direction in this study demonstrates that overflow weirs must carefully balance crest velocity to provide sufficient attract flow without forming an insurmountable barrier, specifically targeting surface-oriented species.

### 5.3. Turbulent Kinetic Energy and Dissipation Rate

The TKE distribution is a critical parameter because complex turbulent flows can cause physiological injuries to fish, such as loss of balance or direction discrimination. Previous studies by [38–42] have mapped TKE in various fishways, generally concluding that Denil fishways exhibit excessively high turbulence levels. Our findings indicate that the TKE in the overflow weir fishway is heavily concentrated in the plunging pool beneath the crest. When comparing our results to the turbulent structures in combined overflow-weir and vertical-slot fishways, it is evident that the overflow weir exerts a more significant influence on turbulence intensity and Reynolds stress in the surface layer than the vertical slot, generating larger-scale eddy structures in the weir flow region, whereas the vertical slot produces smaller-scale eddies. While a higher turbulent dissipation rate indicates better energy dissipation effect—transforming TKE into molecular thermal motion via viscosity—our results highlight a design trade-off: the large-scale eddies and high TKE beneath the weir crest effectively dissipate energy but may disorient fish. Consequently, optimizing an integrated fishway design requires extracting the TKE distribution diagrams, as done in this study, to ensure that the turbulent dissipation rate remains high enough for energy reduction, while the resulting TKE magnitudes do not surpass the behavioral tolerance of the target species, a balance where combined “slot-hole-weir” designs have shown promise by reducing mainstream velocity and turbulence simultaneously [43]. While the present study focuses on characterizing the baseline hydraulics of the overflow weir, ongoing research is exploring innovative configurations in other fishway types to directly enhance fish swimming conditions. For instance, studies on vertical slot fishways [44] demonstrate how geometric modifications can be tailored to reduce turbulence and create more favorable migration corridors, an approach that could inspire future innovations on the basic overflow weir design. This study has clear limitations. Only nine discharge–drop combinations were tested, and additional cases are needed to precisely define the transition flow threshold. Furthermore, validation was performed at the numerical code level rather than with physical overflow weir data. Nevertheless, the consistent hydraulic trends with classic and recent studies confirm that our results are robust for overflow weir fishway hydraulic characterization and structural optimization.

## 6. Conclusion

The flow regime in an overflow weir fishway exhibits distinct two-dimensional characteristics in the x–z plane. As discharge increases, the same model can display plunging, transition, and streaming flows. The critical discharge for transition flow increases with greater pool drop.

For a given upstream discharge, a larger drop between pools increases the likelihood of plunging flow, reduces internal turbulent energy, and expands the range of fish species that can migrate successfully. Therefore, increasing the inter-pool drop is beneficial for fishway operation under constant inflow conditions.

For a given pool drop, a smaller upstream discharge favors plunging flow, reduces turbulent energy in the pool, and accommodates a wider variety of fish. Thus, under constant drop conditions, lower discharges are more suitable for fishway operation.
